# Cross-sectional survey data on the socio-demographic characteristics associated with substance use among internal displaced persons in Maiduguri, Nigeria

**DOI:** 10.1016/j.dib.2023.109875

**Published:** 2023-11-30

**Authors:** Osaro Aigbogun, Deborah Oyine Aluh, Roland Nnaemeka Okoro, Midya Yousefi, Olawole Fawehinmi, Mohammed Sani Abdullahi

**Affiliations:** a Faculty of Business, Curtin University, Miri 98009, Sarawak, Malaysia; b Lisbon Institute of Global Mental Health (LIGMH), NOVA Medical School, NOVA University of Lisbon, 1150-082 Lisboa, Portugal; c Department of Clinical Pharmacy and Pharmacy Administration, University of Maiduguri, Maiduguri, Nigeria; d Wenzhou-Kean University, Wenzhou, Zhejiang Province 325060, China; e Faculty of Business, Economics and Social Development, Universiti Malaysia Terengganu, 21300 Kuala Terengganu, Terengganu, Malaysia; f Department of Management and Marketing, King Fahd University of Petroleum and Minerals, Dhahran, Saudi Arabia

**Keywords:** Drug use, Substance abuse, Substance dependence, DUDIT, IDPs

## Abstract

Regression analysis was carried out to examine the association between certain socio-demographic characteristics and substance use among internally displaced persons (IDPs). Using an adapted version of the Drug Use Disorder Identification Test (DUDIT) instrument, cross-sectional survey data were obtained from 520 IDPs living in three camps located in Maiduguri, Borno state of Nigeria. The data were analyzed using Statistical Package for Social Sciences software version 21.0. Specifically, this article provides data about the participants’ demographic characteristics, the types of substances they use, reasons for using such substances, and the prevalence of substance use. This dataset can offer valuable multivariate information for future research agendas in similar, or closely related study populations. This cross-sectional dataset is also valuable for policymakers who are seeking ways to intervene in the substance use problem, as well as other associated social vices, affecting the vulnerable population of IDPs.

Specifications Table

**Table:** 

Subject	Social Science / Health
Specific subject area	Prevalence of Substance Use, Drug Use Disorder Identification Test, Internally Displaced Persons
Data format	Raw data (unfiltered) in .sav format Analyzed
Type of data	Table
Data collection	A hand-to-hand contact survey was carried out using a questionnaire adapted from the Drug Use Disorder Identification Test (DUDIT) instrument. The data were gathered between November and December 2021, by a group of data collectors proficient in Kanuri and English languages. Eligibility for participation in the study included being an IDP residing in the designated camps, being above 18 years old, being able to provide informed consent, and having proficiency in either the Kanuri or English language. The majority of the individuals participating in the study exhibited low levels of literacy, thereby necessitating the utilization of an interviewer-administered survey.
Data source location	Three IDP camps located in Maiduguri, Borno state of Nigeria 11.8311° N, 13.1510° E
Data accessibility	Repository name: Mendeley Data identification number: 10.17632/9x2xj5h63c1.1 Direct URL to data: https://data.mendeley.com/datasets/9x2xj5h63c/1
Related research article	D.O. Aluh, R. Okoro, O. Aigbogun, Correlates of substance use and dependence among internally displaced persons in Maiduguri, Nigeria. *Journal of Substance Use*. (2023) 1-7. https://doi.org/10.1080/14659891.2023.2213758 [1].

## 1 Value of the Data

• This data provides vital information to better understand the recent increase in substance abuse among IDPs, as well as the underlying factors contributing to this trend.
• Research scholars, policymakers, developmental sector leaders, IDP camp managers/directors, and public sector leaders will find this dataset beneficial through the empirical application of the DUDIT instrument in this vulnerable population, and the insight into the socio-demographic factors that are implicated in the substance abuse problem.
• These data can be reused by other researchers undertaking a comparative study with a similar research population in other geographical locations.
• These data can be reused in future studies investigating or reviewing the impact of social and public sector intervention on the substance use problem in the same population.

## 2 Data Description

Based on a non-probability convenience sampling technique, 200 self-administered questionnaires were distributed to each of the three IDP camps surveyed, making a total number of 600 questionnaires distributed. 520 surveys were returned making a response rate of 86.67 %. The choice of a non-probability convenience sampling technique was due to the unavailability of a sampling frame, and the fact that practicality and ease of access to the IDPs were more essential. Hence, in executing the convenience sampling strategy, the survey respondents were recruited based on their availability and convenience rather than via random techniques.

The data is represented in three tables. Table 1 exhibits a frequency analysis of the survey participants’ socio-demographic features: gender, educational level, marital status, employment status, type(s) of substances used, and number of substances used. Table 2 gives an indication of the most popular reasons for abusing substances among the IDPs: availability of substance (35.1 %, *n* = 108), influence from others (14.3 %, *n* = 44), and having a disease condition (13.96 %, *n* = 43). Table 3 presents inferential data statistics on the associations between socio-demographic features and substance use among the IDPs.

**Table 1:** Sociodemographic characteristics of the study population.

Characteristics	Frequency (N)	Percentage (%)
**Gender**		
Male	124	23.8
Female	396	76.2
**Education**		
Non-formal	383	75.1
Primary	46	9.0
Secondary	61	12.0
Tertiary	20	3.9
**Marital Status**		
Single	101	19.5
Married	327	63.0
Widow	61	11.8
Divorced	30	5.8
**Employment**		
Employed	36	7.1
Unemployed	440	86.3
Retired	7	1.4
Student	27	5.3
**Substances Used**		
Kolanut	242	46.54
Sleeping pills	104	20.0
Bitter kola	81	15.58
Anabolic steroids	67	12.88
Marijuana	31	5.96
Codeine containing syrups	24	4.62
Cocaine	6	1.15
Shoe Polish	6	1.15
Green tea	5	0.96
Pit toilet gas	4	0.77
Tramadol	4	0.77
Glue	3	0.58
Benzhexol	2	0.38
Lizard dung	1	0.19
**Number of substances**		
0	176	33.8
1	182	35.0
2	110	21.2
3	31	6.0
4	16	3.1
5	5	1.0

**Table 2:** Reasons for substance use.

Reasons	Frequencies	Percentage
Availability	108	35.06
Euphoria	44	1.43
Influence from others	44	14.29
Disease condition	43	13.96
Addiction	33	10.71
Enhanced Performance	23	7.47
Unemployment	21	6.82
Pregnancy	2	0.65

**Table 3:** Bivariate and Multivariate associations between socio-demographic characteristics and DUDIT scores of substance dependence.

Variable	Crude estimate	95 % CI	*P*-value	Adjusted estimate	95 % CI	*P*-value
**Gender**						
Female (Ref)	-	-	-	-	-	-
Male	0.696	−1.464 to 2.855	0.527	1.595	−0.813 to 4.003	0.193
**Education**						
Tertiary (Ref)	-	-	-	-	-	-
Secondary	3.155	−2.032 −8.342	0.232	5.138	−0.378 to 9.851	0.069
Primary	3.935	−1.468 to 9.338	0.153	5.326	0.141–10.766	<0.05*
No formal education	6.164	1.780–10.547	0.006**	7.250	2.228–11.480	<0.01**
**Marital status**						
Divorced (Ref)	-	-	-	-	-	-
Widowed	−3.353	−7.386 to 0.679	0.103	−4.669	−8.509 to (−0.830)	0.017*
Married	−5.844	−9.424 to (−2.264)	0.001**	−6.696	−10.089 to (−3.303)	<0.01**
Single	−3.527	−7.548–0.494	0.085	−2.625	−6.746 to 1.496	0.211
**Employment**						
Student (Ref)						
Retired	4.452	−5.315 to 4.220	0.370	4.585	−4.613 to 13.783	0.327
Unemployed	9.483	5.274–13.692	<0.001**	7.777	3.442–12.111	<0.01**
Employed	8.013	2.764–13.262	0.003*	7.749	2.389–13.108	<0.01**
**Number of substances used**						
5 (Ref)	-					
4	−6.333	−16.151 to 3.484	0.205	−2.886	−12.101 to 6.329	0.538
3	−10.481	−19.928 to (−1.035)	0.030*	−6.679	−15.571 to 2.213	0.140
2	−12.310	−21.404 to (−3.216)	0.008**	−9.975	−18.487 to (−1.462)	<0.05*
1	−14.455	−23.498 to (−5.411)	0.002**	−10.954	−19.439 to (−2.468)	<0.05*
None	−5.167	−19.337 to 9.004	0.474	−1.441	−14.873 to 11.992	0.833
**Age**	0.090	0.025–0.155	0.007**	0.060	−0.015 to 0.135	0.116
**Time spent in camp**	0.029	−0.440 to 0.499	0.903	−0.151	−0.589 to 0.287	0.497

Level of significance (*p < 0.05, **p < 0.01).

The raw unfiltered data, questionnaire (in English and Kanuri), consent form, and approval to conduct the survey are available as supplemental files through the Mendeley Data repository [2].

Based on the descriptive statistical results generated by IBM SPSS, as shown in Table 1, the majority of the study population consisted of females (76.2 %, *n* = 396), with 75.1 % (*n* = 383) lacking any formal education. Moreover, the majority of them (86.3 %, *n* = 440) were unemployed. The study population reported using bitter kola (15.6 %, *n* = 81), sleeping pills (20.0 %, n – 104), anabolic steroids (12.9 %, *n* = 67), and kolanut (46.5 %, *n* = 242) most frequently. A total of 162 participants (31.3 %) used more than one substance concurrently, with 5 individuals (1 %) using five substances concurrently.

Table 2 reveals the output from the descriptive statistics analysis carried out using IBM SPSS. It shows that the most popular reasons for using substance were availability of substance (35.06 %, *n* = 108), influence from others (14.29 %, *n* = 44) and having a disease condition (13.96 %, *n* = 43).

Table 3 shows the output from the univariate and multivariate linear regression analyses carried out using IBM SPSS with the total dependence score as the outcome variable. According to the Nagelkerke R2, this model explained 20.2 % of the variation in the DUDIT scores among the internally displaced people surveyed. Individuals who were internally displaced and lacked formal education scored 6.164 points higher on the DUDIT than those who had completed higher education (CI: 1.780–10.547, *P* < 0.01). The total DUDIT scores increased gradually as the number of substances used increased, albeit this trend lost significance when there were three or four substances used as opposed to five. After adjusting for other characteristics, individuals who used one substance concurrently had 10.954 lower DUDIT scores than those who used five substances concurrently (CI: −19.439−(−2.468), *P* =< 0.05). According to the DUDIT scores, education, marital status, employment status, and the number of substances used were still significantly linked to substance dependence.

## 3 Experimental Design, Materials and Methods

**Fig. 1:**
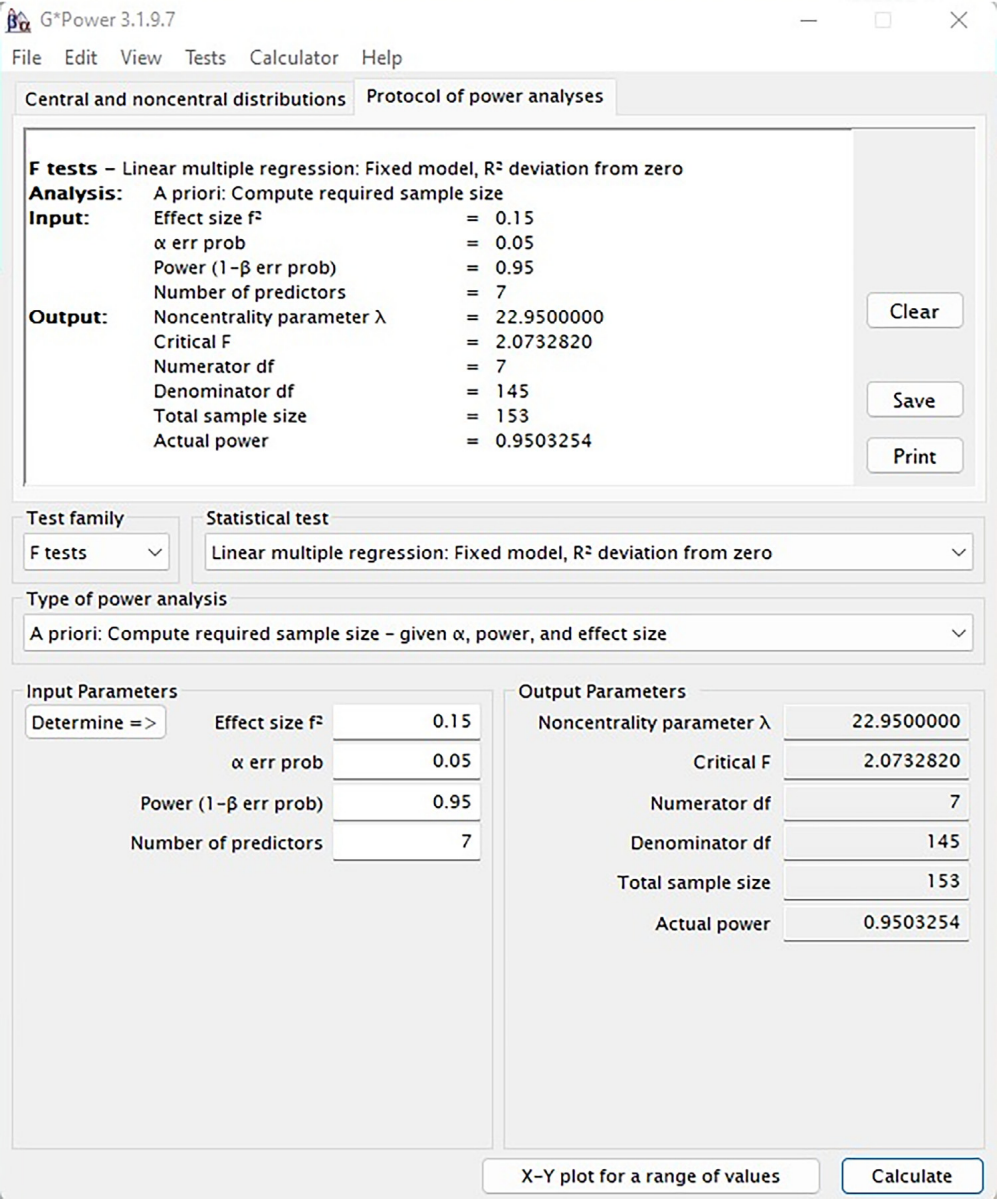
Sample size determination via power analysis.

Therefore, a priori power analysis was carried out using the G*Power software (version 3.1.9) to determine a sample size that guaranteed the robustness of the data [3]. After entering the desired statistical power (1 – β = 0.95), effect size (f2 = 0.15), significance level (*α* = 0.05), and total number of predictors (seven in this case), the findings showed that a sample size of 153 is optimal for a regression-based statistical analysis (Fig. 1).

In the cross-sectional survey conducted between November and December 2021, data were collected from a conveniently recruited sample residing in three IDP camps located in Maiduguri, Borno state of Nigeria. Through the Borno State Emergency Management Agency, the Borno State Ministry of Health granted permission for this study to be conducted in the three IDP camps surveyed. After they were informed of the study's goals, the survey participants gave their informed consent. They received guarantees of the study’s data confidentiality and anonymity, as well as the option to withdraw their permission or participation at any time.

The research instrument (survey questionnaire) consisted of three sections. The first section gathered data regarding the sociodemographic characteristics of the individuals involved in the survey. The second section placed emphasis on the DUDIT, wherein modifications were made by incorporating substances that were easily accessible at the study location into the existing list of substances available in the original version of DUDIT [4]. The objective of the third section of the questionnaire was to identify the underlying factors that drive individuals to engage in substance use. The DUDIT is a psychometric instrument of eleven questions that employ Likert scale anchors. Its primary objective is to assess the level of substance dependence. Questions 10 and 11 are assessed using a scoring system that assigns values of 0, 2, or 4, whereas questions 1 through 9 are evaluated on a scale ranging from 0 to 4 [4]. The screening criteria for males is 6, whilst for females it is 2. The DUDIT has been empirically validated as a reliable and valid instrument for assessing drug usage [4,5]. For this study, the DUDIT instrument underwent a process of translation into the Kanuri language, followed by a back-translation into English, with the consent of its developers. Kanuri, the native language of Borno State, is predominantly spoken by the IDPs. The translation underwent a thorough evaluation conducted by linguistic specialists affiliated with the Department of Linguistics at the University of Maiduguri in Borno State. The DUDIT was created as a complementary tool to the AUDIT (Alcohol Use Disorders Identification Test) in order to identify persons who may be experiencing issues connected to drug/substance use. The decision to employ the DUDIT in this study was deliberate due to the majority Muslim composition of the study location, which adheres to a prohibition on the consumption of alcoholic beverages. The DUDIT has demonstrated efficacy in identifying drug-related issues within specific clinical populations and has been proposed as a valuable tool for public health surveys [5,6].

The sampled population was characterized using descriptive analysis. The instrument cut-off scores were used to determine the prevalence of substance use and dependence. Probable drug dependency on one or more substances is indicated by scores of 25 and above for both genders. The outcome variable used in these analyses was the total dependence score, which was subjected to both univariate and multivariate linear regression. *P*-values of 0.05 or less were regarded as significant.

## Limitations

According to the survey participants’ demographic profile, women made up over 75 % of the study sample, and many of them had no formal education. The fact that there are more women than men living in the IDP camps can be explained by the fact that terrorists mostly target men to murder or recruit as combatants. Moreover, a significant percentage of the study population lack formal education, which may be attributed to the overall low literacy rate in Northern Nigeria where the study was conducted. Despite these limitations, the data obtained using the DUDIT, an instrument that is proven to be reliable and valid, presents a factual picture that underscores the need for intervention in this study population

## Ethics Statement

Approval for conducting this study was granted by the Borno State Ministry of Health through the Borno State Emergency Management Agency (see supplemental files). As this was a voluntary, anonymous, non-experimental survey, no ethical clearance was necessary in accordance with the Item b of Section B (Exemption) of the National Code of Health Research Ethics of Nigeria [7]. Nonetheless, in order for participants to complete the anonymous survey, their informed consent was obtained. No personal information, such as the names, phone numbers, or email addresses of the responders, were solicited; and the data was anonymized. They also received guarantees about their privacy and confidentiality as well as the option to revoke their consent at any time.

## Credit Author Statement

**Osaro Aigbogun**: Data curation, Writing, Original draft preparation. **Deborah Aluh:** Conceptualization, Methodology, Analysis. **Roland Okoro**: Investigation, Survey coordination, Supervision. **Midya Youseffi:** Writing- Reviewing and Editing. **Olawole Fawehinmi:** Data curation and Original draft preparation. **Mohammed Sani Abdullahi**. Data curation

## Data Availability

Socio-demographic Factors linked to Substance use among Internally Displaced Persons in Maiduguri, Nigeria: Insights from Cross-Sectional Survey Data (Original data) (Mendeley Data)
